# Violent Experiences and Patterns of Firearm Ownership From Childhood to Young Adulthood

**DOI:** 10.1001/jamanetworkopen.2023.36907

**Published:** 2023-10-18

**Authors:** Josie Caves Sivaraman, Guangyu Tong, Michele Easter, Jeffrey Swanson, William Copeland

**Affiliations:** 1 RTI International, Research Triangle Park, North Carolina; 2 Department of Biostatistics, Yale School of Medicine, New Haven, Connecticut; 3 Department of Psychiatry and Behavioral Sciences, Duke University, Durham, North Carolina; 4 School of Medicine, University of Vermont, Burlington

## Abstract

**Importance:**

Young adults in their 20s are at high relative risk for self- and other-directed firearm injury, but little is known about gun access patterns for this group.

**Objective:**

To describe the longitudinal patterns of firearm access from childhood to young adulthood and to estimate whether violence experienced as a child or as an adult is associated with gun ownership in young adulthood.

**Design, Setting, and Participants:**

The Great Smoky Mountains Study included participants from 11 contiguous, mostly rural counties in the Southeastern US. The first wave was completed in 1993 and the most recent in 2019. Periodic survey data were gathered in adolescence through participants’ late 20s. In 2023, adjusted Poisson regression with incident rate ratios (IRRs) and 95% CIs were used to estimate associations between violence and gun ownership in young adulthood in 3 age cohorts from the original sample.

**Exposures:**

Violent experiences in childhood (bullying, sexual and physical abuse, violent events, witnessing trauma, physical violence between parents, and school/neighborhood dangerousness) or adulthood (physical and sexual assault).

**Main Outcomes and Measures:**

Initiating gun ownership was defined as no gun access or ownership in childhood followed by gun ownership at age 25 or 30 years. Maintaining gun ownership was defined as reporting gun access or ownership in at least 1 survey in childhood and ownership at age 25 or 30 years.

**Results:**

Among 1260 participants (679 [54%] male; ages 9, 11, and 13 years), gun access or ownership was more common in childhood (women: 366 [63%]; men: 517 [76%]) than in adulthood (women: 207 [36%]; men: 370 [54%]). The most common longitudinal pattern was consistent access or ownership from childhood to adulthood (373 [35%]) followed by having access or ownership in childhood only (408 [32%]). Most of the violent exposures evaluated were not significantly associated with the outcomes. Being bullied at school was common and was associated with reduced ownership initiation (IRR, 0.76; 95% CI, 0.61-0.94). Witnessing a violent event was significantly associated with increased probability of becoming a gun owner in adulthood (IRR, 1.24; 95% CI, 1.03-1.49).

**Conclusions and Relevance:**

In this cohort study, gun ownership and access were transitory, even in a geographic area where gun culture is strong. Early adulthood—when the prevalence of gun ownership was relatively low—may represent an opportune time for clinicians and communities to provide education on the risks associated with firearm access, as well as strategies for risk mitigation.

## Key Points

**Question:**

How do patterns of firearm access change from childhood to young adulthood, and how are violent experiences associated with either initiating or maintaining gun ownership or access?

**Findings:**

In this cohort study of 1260 participants in the Southeastern US, access to firearms declined from childhood to young adulthood. Most childhood violent experiences were not significantly associated with gun ownership in young adulthood.

**Meaning:**

Although young adults are at increased risk of gun violence compared with other age groups, the findings in this study demonstrated reduced ownership and access in young adulthood compared to earlier in life, with no increased likelihood of adult ownership among children who experienced most types of violence.

## Teaser

This cohort study evaluates associations of gun ownership in adulthood with violence experienced and firearm access in childhood.

## Introduction

Firearm-related mortality in the US is concentrated among young people. In 2020, more than 15 000 individuals younger than 30 years died as the result of a gunshot, accounting for about one-third of the nation’s annual gun deaths. Among US adults aged 20 to 29 years, the rate of gun-involved homicides and suicides increased by 37% and 14% respectively in that year.^1^ Numerous studies of gun violence at the individual^2,3,4,5,6,7,8,9^ and ecological^10,11,12,13,14,15,16^ levels have found that the presence of a firearm is associated with an approximately 4-fold increase in risk of suicide death and a 2-fold increase in the risk of homicide death. Young adulthood is uniquely marked by a considerable increase in the prevalence of injurious behavior and broadened access to lethal means through the extension of the legal right to purchase and possess firearms, especially handguns. Consequently, understanding which young adults decide to initiate or maintain gun ownership and access, as well as what childhood characteristics and experiences might predict those decisions, can inform harm-reduction approaches to firearm violence.

Recent cross-sectional survey data revealed that 43% of respondents aged 18 to 29 years lived in a household with a gun, including 27% who reported that they personally owned a gun.^17^ Gun ownership is most prevalent in the South and in rural areas, and among men (39%) and White people (36%).^17^ Being raised around guns has also been shown to predict future ownership.^17,18,19^ Though the proportion of US gun-owning households declined from a national average of 45% in 1980 to about one-third of households in the mid-2000s,^20^ among White families with young children, handgun ownership increased from 25% to 38%.^16^ This trend was associated with an increase in firearm-related mortality among 1- to 5-year-olds.^16^

The proportion of gun owners who cite personal protection as a primary motivation for ownership has grown from 48% to 76% over the past 2 decades, signaling a growing public acceptance of firearms as an appropriate and empowering strategy to address fear of external violence.^17,18^ Recently, firearm marketing campaigns have targeted women in this vein, with slogans like “God Created Man and Woman, But Samuel Colt Made Them Equal.”^21^ At the individual level, though, evidence regarding associations between personal experiences of violence and gun ownership or gun carrying is mixed,^22,23,24,25,26^ with positive associations found between experiencing violence and firearm carrying behavior among youths.^22,23,27,28,29,30^ An understanding of how lasting the effects of violent experiences are on gun access has been hindered by a lack of longitudinal, individual-level data on firearms. Moreover, to our knowledge, no study differentiates between the population of individuals who initiate gun ownership in young adulthood and those who maintain their gun access or ownership from childhood. Understanding these 2 categories of gun owners can inform individualized approaches to promoting gun safety and reduce gun violence. Research relating to gun access and comparing adolescent to adult populations was a stated priority in a recent scoping review of this topic.^23^

The present study makes use of unique, longitudinal cohort data from a part of the Southeastern US that is mostly rural (with 1 urban center included, population approximately 100 000) and characterized by a strong culture of gun ownership.^31^ Where the study was conducted there is no minimum age for the possession of rifles and shotguns, and minors are also allowed to possess handguns under the supervision of an adult. We aimed to describe the longitudinal patterns of firearm access and ownership from childhood to young adulthood and estimate how violence experienced in childhood and adulthood are associated with both maintaining and initiating gun ownership in young adulthood. We hypothesized that with greater exposure to childhood violence, young adults would be more likely to maintain (vs desist from) gun ownership if they owned or had access to guns in childhood or to initiate (vs continue to abstain from) gun ownership if they did not own or have access to guns in childhood.

## Methods

The Great Smoky Mountains Study (GSMS)^32,33^ began in 1993 and collected annual survey data on 3 community-representative cohorts of children aged 9, 11, and 13 years. The most recent wave was completed in 2019, providing 26 years of follow-up. Participants were residents of 11 contiguous, mostly rural counties in the Southeastern US. Because the intent of the study was to evaluate the natural history of psychiatric disease, particularly among American Indian youth, the study oversampled American Indian children and children who were designated high risk on the Child Behavior Checklist. Surveys were completed on an almost annual basis through age 16 years for all 3 cohorts, and then participants were surveyed in adulthood at ages 19, 21, 25, and 30 years. The data collected from these surveys focused on a wide breadth of topics related to behavioral health, including gun ownership and access. Before interviews, all participants and their parents signed informed consent forms. The study was approved by the Duke University Medical Center ethical review board. This report follows the Strengthening the Reporting of Observational Studies in Epidemiology (STROBE) reporting guideline.

### Gun Ownership and Access

Childhood gun ownership or access was assessed in structured interviews with parents and children through age 16 years and was coded hierarchically from own gun, access to gun (not owner), gun in home but no access, and no gun in home.^34^ Gun ownership or access was endorsed if reported by either the parent or the child. To be in the final group, the person must never have endorsed any gun access or ownership during any of the childhood surveys. Adult gun ownership and access was assessed at ages 25 and 30 years. Data at ages 19 and 21 years were not included in either the childhood or adult gun coding scheme because respondents were frequently living with their families of origin.

We further simplified the above coding scheme into longitudinal firearm ownership and access patterns. Never owned referred to people who did not have any ownership or access. Adult-only owners were those who did not have ownership or access in childhood but became owners in adulthood. Childhood-only access or ownership included those with ownership or access in childhood only who did not own or have in-home access to guns at ages 25 and 30 years, and consistent owners were those who had access or ownership at some point in childhood and adulthood. People who had access but were not owners in adulthood were dropped from the statistical models because it was unclear the degree to which these individuals actively chose to have gun access. This group was included in sensitivity analyses.

### Violence Measures

Exposure to violent experiences in childhood was based on structured interviews that assessed whether the participant experienced bullying, sexual abuse, physical abuse, or other violent events. These were assessed using the life events module of the Child and Adolescent Psychiatric Assessment Interview^35^ with construction and psychometric properties described elsewhere.^36^ In addition, we included measures that assessed whether the participant had witnessed trauma (ie, experiences in which others’ lives were in danger) or been exposed to physical violence between their parents. Lastly, both participants and parents were asked whether they felt safe in their neighborhood. All of these were assessed as lifetime experiences at each survey wave except for school bullying and neighborhood safety, which referred to the past 3 months. A total count variable (0 to 7) was created to reflect the total number of different types of violence each participant experienced in childhood. In adulthood, exposure to physical or sexual assault was defined consistently with the childhood definitions and endorsed if participants reported having the experience at 19 years or older.

### Covariates

Demographic data were drawn from baseline interviews and included sex (male and female) and race (American Indian, Black, and White), reflecting the US Census categories at the time. Categories were based on US Census categories at the time. Race was included in the analysis because of longstanding demographic differences in gun ownership prevalence and exposure to violence. Low socioeconomic status (yes or no) in childhood was indicated by the presence of 2 or more of the following: low family income, low parental educational attainment, or low occupational status. Urbanicity was based on county Rural-Urban Continuum Codes and was coded dichotomously (yes or no). In addition, we included 3 indices of childhood vulnerability: environmental vulnerability (eg, family poverty, unemployment, and single-parent households), family dysfunction (eg, violence between parents, frequent parental arguments, and use of poor parenting strategies [a composite of related Child and Adolescent Psychiatric Assessment codes]), and parental challenges (eg, parental mental health, substance use, and criminal or legal problems).

### Statistical Analysis

We described the frequency of each longitudinal firearm ownership pattern and related demographic characteristics, risk factors, and violent experiences, stratified by sex. We conducted a series of Poisson regression analyses to estimate associations between reported experiences of violence and initiating gun ownership (comparing 2 groups who did not have access to guns in childhood: adult-only owners vs those who never had access or ownership) and, separately, between experiences of violence and maintaining gun ownership (comparing 2 groups who did have access to guns in childhood: consistent owners to those with childhood access or ownership only). Poisson regression yields incident rate ratios (IRRs), which are less biased approximations of risk ratios than odds ratios when common outcomes are being evaluated. Each model measured a specific childhood or adulthood violence exposure or the count of childhood violence exposure types. We also included an interaction term in the models to test the joint association of violent experience and sex. Sampling weights were reflected in the analyses. We report adjusted IRRs and 95% CIs. As these analyses are mostly exploratory, for the main associations and interaction terms we considered statistical significance to be *P* < .05.

### Missing Data

**Figure 1.**
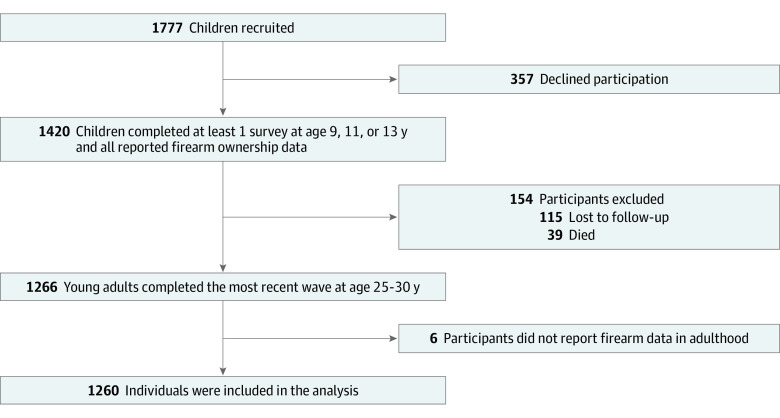
Flow Diagram

Of the 1381 living participants in the final wave, 1260 were followed up at ages 25 or 30 years and had complete firearm data (loss to follow-up was 8.8%) (Figure 1). People without childhood gun access or ownership had higher attrition, perhaps related to other characteristics (eg, urbanicity and socioeconomic status) that made them more likely to move out of the region. This did not introduce a selection bias because the analyses divided the participants based on childhood access and made comparisons within those groups.

## Results

### Describing Patterns of Gun Ownership

**Figure 2.**
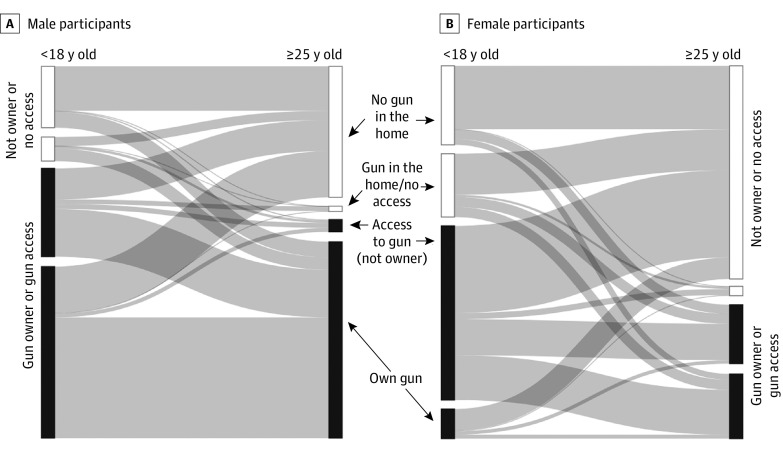
Gun Access and Ownership Transitions Between Childhood and Adulthood

The study included 1260 participants (581 women [46%] and 679 men [54%]; ages 9, 11, and 13 years at baseline) with complete firearm ownership and access data through at least age 25 to 30 years. In terms of race, 318 participants (25%) were American Indian, 81 (6%) were Black, and 861 (68%) were White. As measured in childhood, 734 (58%) lived in rural areas and 499 (40%) had low socioeconomic status. Figure 2 depicts transitions in gun access and ownership between childhood and adulthood by sex. Most participants of either sex had gun ownership or access as children (girls: 366 of 581 [63%]; boys: 517 of 679 [76%]). In adulthood, most men (370 of 679 [55%]) continued to have gun ownership or access compared with a minority of women (207 of 581 [36%]). Overall, 195 of 517 men from households with gun ownership or access (27%) desisted ownership in adulthood, and 48 of 162 (22%) made the opposite transition. Although most female participants came from homes with gun access, far fewer reported owning guns during childhood than their male counterparts. Half of women who grew up in a household with gun ownership or access transitioned to a living situation without guns (213 of 366 [58%]), and 54 of 217 (25%) made the move from a household without guns to gun ownership.

Participants who had access but not ownership in adulthood were excluded from analytic models (n = 140). It was relatively uncommon to become a gun owner for the first time in adulthood (64 of 1120 [7%]); Table 1 percentages reflect population weights. In the combined sample of men and women, the most common developmental pattern in gun ownership was consistent access (ie, owning or having access to a gun in childhood and owning a gun in adulthood; 373 of 1120 [35%]), followed by those who owned or had access to guns in childhood only (408 of 1120 [32%]) and those who never owned guns (275 of 1120 [26%]).

**Table 1.** Description of Longitudinal Patterns of Gun Access and Ownership

Variable	No (%)		*P* value	No (%)		*P* value
	Never owned or had access	Adult-only owners (new access)		Childhood access only	Consistent access	
Total No.	275 (25.9)	64 (7.1)	NA	408 (31.9)	373 (35.1)	NA
Demographic characteristics						
Sex						
Female	161 (66.3)	23 (36.0)	<.001	213 (58.1)	80 (22.3)	<.001
Male	114 (33.7)	41 (64.0)		195 (42.0)	293 (77.7)	
Race^a^						
American Indian	55 (2.8)	7 (1.3)	.002	142 (5.9)	76 (3.8)	.002
Black	48 (18.6)	6 (3.4)		20 (6.8)	3 (2.1)	
White	172 (78.6)	51 (95.3)		246 (87.4)	294 (94.1)	
Residence						
Urban	170 (75.6)	37 (60.8)	.01	150 (56.9)	110 (40.5)	<.001
Rural	105 (24.4)	26 (39.2)		256 (43.1)	260 (59.5)	
Low socioeconomic status						
Yes	119 (26.7)	25 (16.4)	.06	178 (34.8)	125 (26.6)	.01
No	156 (73.3)	37 (83.6)		228 (65.2)	248 (73.4)	
Childhood vulnerability, mean (SD)						
Environmental vulnerability	1.7 (1.7)	1.3 (1.5)	.04	1.7 (1.5)	1.6 (1.3)	.32
Family dysfunction	0.5 (0.5)	0.5 (0.6)	.80	0.5 (0.5)	0.5 (0.4)	.55
Family history of drug use/crime/mental disorder	0.7 (0.8)	0.8 (1.0)	.41	0.6 (0.6)	0.6 (0.6)	.07

Abbreviation: NA, not applicable.

^a^ Race was gathered via self-report according to categories defined by the US Census and included in the study because of longstanding demographic differences in gun ownership prevalence and exposure to violence.

### Associations of Violence Exposure With Gun Access or Ownership

Table 2 displays results of the analysis to test whether exposure to violence was associated with initiating gun ownership for the first time in adulthood. The most common event types were violent events, witnessing trauma, and being bullied at school. Of the childhood exposures, only witnessing a violent event was significantly associated with increased probability of becoming a gun owner in adulthood (IRR, 1.24; 95% CI, 1.03-1.49), and being bullied was associated with a lower likelihood of transitioning to adult gun ownership (IRR, 0.76; 95% CI, 0.61-0.94). Sex-specific estimates revealed that the negative association with bullying was stronger among male participants, although there was no statistically significant interaction for sex (eTable 1 in Supplement 1). The effect size estimate for sexual abuse was similar to that of being bullied at school, but this result was not statistically significant (IRR, 0.76; 95% CI, 0.56-1.04). Exposures in adulthood were not statistically significantly associated with becoming a gun owner in adulthood.

**Table 2.** Associations Between Violent Experiences and Becoming a Gun Owner in Adulthood (Adult-Only Access) vs Never Owning a Gun^a^

Violent experience	No. (%)		IRR for adult-only ownership (95% CI)	*P* value
	Prevalence: never owned or had access	Prevalence: adult-only owners		
Childhood				
Bullied at school	87 (29.4)	25 (13.0)	0.76 (0.61-0.94)	.01
Sexual abuse	51 (13.6)	4 (2.2)	0.76 (0.56-1.04)	.09
Physical abuse	41 (11.0)	9 (4.9)	0.94 (0.68-1.30)	.71
Violent event	146(54.6)	32(58.9)	1.11 (0.92-1.34)	.27
Witnessed trauma	104 (32.3)	24 (42.7)	1.24 (1.03-1.49)	.03
Physical violence between parents	24 (10.9)	6(3.3)	0.90 (0.61-1.33)	.59
Dangerous school or neighborhood	38 (9.4)	10 (6.3)	0.89 (0.61-1.31)	.55
Count of violence types, mean (SD)	1.53 (1.37)	1.36 (1.23)	0.98 (0.90-1.07)	.70
Adulthood				
Physical assault	45 (11.6)	21 (14.7)	1.14 (0.84-1.53)	.40
Sexual assault	29 (9.4)	17 (10.8)	1.11 (0.78-1.58)	.55

Abbreviation: IRR, incident rate ratio.

^a^ All models adjusted for race, sex, cohort, urbanicity, low socioeconomic status, and childhood vulnerabilities.

Table 3 displays results of the analysis to test associations between exposure to violence and whether those who owned or had access to guns in childhood continued to own guns in adulthood. Again, experiencing a violent event, witnessing trauma, and being bullied were common exposures. None of the associations evaluated in this set of models were statistically significant. Similarly, exposure to physical and sexual assault in adulthood were not associated with continued gun ownership, and there were no significant interactions with sex in these models (eTable 2 in Supplement 1). Sensitivity models that included adults with firearm access or ownership in the models yielded similar results to those reported in the main analysis, both for initiating firearm ownership (eTable 3 in Supplement 1) and maintaining firearm ownership (eTable 4 in Supplement 1).

**Table 3.** Associations Between Violent Experiences and Gun Ownership in Young Adulthood (Consistent Access) vs Having Childhood Access Only^a^

Violent experience	No. (%)		IRR for consistent access (95% CI)	*P* value
	Prevalence: consistent access	Prevalence: childhood access -only		
Childhood				
Bullied at school	106 (26.2)	123 (26.9)	0.99 (0.90-1.10)	.91
Sexual abuse	30 (7.5)	44 (14.6)	0.94 (0.82-1.09)	.44
Physical abuse	43 (8.7)	77 (18.9)	0.88 (0.77-1.02)	.08
Violent event	230 (53.2)	249(49.2)	1.02 (0.94-1.12)	.60
Witnessed trauma	142 (25.3)	164 (35.0)	0.97 (0.88-1.06)	.49
Physical violence between parents	42 (8.3)	29 (6.9)	1.03 (0.86-1.23)	.76
Dangerous school or neighborhood	30 (5.2)	50 (7.1)	0.96 (0.79-1.18)	.72
Count of violence types, mean (SD)	1.19 (1.15)	1.56 (1.28)	0.97 (0.93-1.01)	.13
Adulthood				
Physical assault	85 (15.7)	78 (23.9)	1.05 (0.93-1.18)	.43
Sexual assault	37 (9.7)	46 (10.8)	1.11 (0.94-1.31)	.21

Abbreviation: IRR, incident rate ratio.

^a^ All models adjusted for race, sex, cohort, urbanicity, low SES, and childhood vulnerabilities.

## Discussion

This cohort study made use of unique longitudinal data to characterize patterns of gun ownership from early adolescence through young adulthood in an area of the country where gun culture is often considered pervasive. Indeed, most study participants not only grew up in households with guns, but most could access those firearms in childhood, and half of male participants personally owned guns while still under age 18. However, despite the high prevalence of gun access in childhood, we identified substantial variation in gun ownership during the transition period from childhood to young adulthood, particularly among women. This period in life is one of self-exploration and identity formation, when individuals select the values and traditions that they will carry forward from childhood and those that will differentiate them from the previous generation.^37^ Overall, both male and female participants were less likely to have gun access as young adults than as children.

The decline in gun ownership or access in early adulthood may be intentional, situational (eg, leaving one’s childhood home), or both, but it is especially relevant to harm reduction efforts because it suggests that firearm access during this decade of life may be uniquely subject to influence. Although most discussions about guns in health care settings are targeted to parents of at-risk children (ie, interventions such as Safety Check^38^ and Counselling on Access to Lethal Means^39^), addressing firearm access during this critical and formative period is worthy of further investigation for at least 2 reasons. First, young adulthood is a time of high personal risk of firearm injury, as discussed previously. Second, it is a time when many people start their own families and establish norms for the next generation. Evidence that firearm access is malleable during this decade may encourage individual and community-level messaging that frames safe storage and child access limits as appropriate and empowering methods of advancing personal, family, and community safety.

In our analysis of the association between childhood violent experiences and firearm ownership in adulthood, the number of statistically significant associations identified was close to what might have been expected by chance when running this number of models. Because these are population-level associations and because they are subject to unmeasured and potentially confounding influences, we cannot draw causal conclusions. With that caution, this study provides some evidence that people with certain childhood experiences of direct violence (as opposed to witnessed violence) may have reduced likelihood of firearm ownership in adulthood. Although exposures like being bullied were common, firearm ownership declined over time. This was contrary to our hypothesis, particularly considering previous research that found associations between adverse childhood experiences and more risky behavioral outcomes in adulthood.^40^ However, it is consistent with past findings that interpersonal firearm violence tends to occur frequently in concentrated social networks rather than infrequently across the wide swath of people who grew up experiencing violence.^41^ Each individual’s unique socialization likely drives their responses to experiences of violence and their firearm ownership decisions.

Indeed, our null findings suggest that even in regions where gun ownership is highly prevalent, individuals likely have a diversity of experiences that impact their attitudes and practices surrounding firearms, which the research community has only begun to describe and understand. As the threat of gun violence continues to change and becomes an aspect of daily life for many school-aged people and young adults, a research agenda is needed that specifically addresses how individuals interpret these effects. Translational research can identify avenues and partnerships for public health professionals and clinicians to effectively integrate firearm harm reduction strategies, building a nuanced approach to firearm violence prevention.

### Strengths and Limitations

To our knowledge, this is the first study to longitudinally characterize firearm ownership in the same individuals followed from early adolescence through young adulthood. There were several limitations to our approach. The sample, which was drawn from a mostly rural area of the Southeastern US, is characterized by high levels of gun ownership and may not be generalizable to the rest of the US. However, this sample did allow for rich data collection, with nearly annual capture of exposures during adolescence and the achievement of extremely high retention. Recent events, such as the COVID-19 pandemic and civil unrest, are also known to have increased gun ownership and access in the country overall. Though national data suggest that the number of new gun owners increased by an estimated 2.9% during that period, our study does not capture this period.^42^

## Conclusions

In this cohort study of longitudinal patterns of firearm ownership, access from youth to adulthood differed for men and women, but gun access was somewhat reduced in young adulthood for both. We also found that most violent experiences from early life were not associated with either initiating or maintaining gun ownership, and where there was evidence, the direction of association was inconsistent. Early adulthood may be a time when people are still formulating their opinions about whether to engage in firearm ownership or access, and research that further explores these themes (within and across sex) and how clinicians can provide education and strategies for firearm risk mitigation to this population is warranted.

## Notes

Supplement 1. **eTable 1.** Sex stratified results for the associations between violent experiences and becoming a gun owner in adulthood (adult-only owner) versus remaining a never owner **eTable 2.** Sex stratified associations between violent experiences and remaining a gun owner in young adulthood (consistent owner) versus being a childhood-only owner **eTable 3.** Associations between violent experiences and becoming a gun owner in adulthood (adult-only owner) versus remaining a never owner sensitivity analysis: the “adult-only ownership” category includes people who owned or had access to guns in adulthood **eTable 4.** Associations between violent experiences and remaining a gun owner in young adulthood (consistent owner) versus being a childhood-only owner sensitivity analysis: the “consistent owners” category includes who owned or had access to guns in adulthood

Supplement 2.
Data Sharing Statement
