# Supplementary material for: Violent Experiences and Patterns of Firearm Ownership From Childhood to Young Adulthood
Source: JAMA Netw Open. 2023 Oct 18;6(10):e2336907. doi: 10.1001/jamanetworkopen.2023.36907 (PMC10585415; doi:10.1001/jamanetworkopen.2023.36907)
Supplement: Supplement 1. — eTable 1. Sex stratified results for the associations between violent experiences and becoming a gun owner in adulthood (adult-only owner) versus remaining a never owner eTable 2. Sex stratified associations between violent experiences and remaining a gun owner in young adulthood (consistent owner) versus being a childhood-only owner eTable 3. Associations between violent experiences and becoming a gun owner in adulthood (adult-only owner) versus remaining a never owner sensitivity analysis: the “adult-only ownership” category includes people who owned or had access to guns in adulthood eTable 4. Associations between violent experiences and remaining a gun owner in young adulthood (consistent owner) versus being a childhood-only owner sensitivity analysis: the “consistent owners” category includes who owned or had access to guns in adulthood [file jamanetwopen-e2336907-s001.pdf]

Supplemental Online Content

Caves Sivaraman J, Tong G, Easter M, Swanson J, Copeland W. Violent experiences and firearm ownership from childhood to young adulthood. *JAMA Netw Open*. 2023;6(10):e2336907. doi:10.1001/jamanetworkopen.2023.36907

eTable 1. Sex stratified results for the associations between violent experiences and becoming a gun owner in adulthood (adult-only owner) versus remaining a never owner

eTable 2. Sex stratified associations between violent experiences and remaining a gun owner in young adulthood (consistent owner) versus being a childhood-only owner

eTable 3. Associations between violent experiences and becoming a gun owner in adulthood (Adult-only Owner) versus remaining a Never Owner sensitivity analysis: the "adult-only ownership" category includes people who owned or had access to guns in adulthood

eTable 4. Associations between violent experiences and remaining a gun owner in young adulthood (consistent owner) versus being a childhood-only owner sensitivity analysis: the "consistent owners" category includes who owned or had access to guns in adulthood

This supplemental material has been provided by the authors to give readers additional information about their work.

© 2023 Caves Sivaraman J et al. *JAMA Network Open.*

eTable 1. Sex stratified results for the associations between violent experiences and becoming a gun owner in adulthood (adult-only owner) versus remaining a never owner

	Male					Female				p-value for interaction
	**IRR**	**LCI**	**UCI**	**p value**		**IRR**	**LCI**	**UCI**	**p value**	
**School Bully**	0.71	0.52	0.98	0.0351		0.84	0.60	1.18	0.3193	0.6526
**Sexual Abuse**	0.66	0.41	1.06	0.0832		0.88	0.56	1.38	0.5703	0.4713
**Physical Abuse**	1.15	0.67	1.97	0.6107		0.88	0.57	1.34	0.5465	0.6551
**Violent Event**	1.13	0.85	1.50	0.4073		1.09	0.85	1.41	0.4957	0.9102
**Witness Trauma**	1.22	0.92	1.61	0.1713		1.24	0.95	1.61	0.1171	0.9161
**Physical Violence btw. Parents**	0.80	0.38	1.65	0.5412		0.97	0.58	1.62	0.9127	0.8042
**Dangerous sch/neighbor**	0.95	0.52	1.75	0.8810		0.85	0.50	1.46	0.5572	0.6477
**Count of violence types mean/sd**	0.97	0.84	1.11	0.6501		1.02	0.90	1.15	0.7665	0.8729
**Adulthood Victim(18+)**										
**Physical assault**	0.97	0.52	1.83	0.9295		1.18	0.84	1.67	0.3371	0.5883
**Sexual assault**	1.30	0.56	3.04	0.5394		1.09	0.71	1.68	0.6914	0.6988

All models adjusted for race, sex, cohort, urbanicity, low SES, and childhood vulnerabilities

© 2023 Caves Sivaraman J et al. *JAMA Network Open.*

eTable 2. Sex stratified associations between violent experiences and remaining a gun owner in young adulthood (consistent owner) versus being a childhood-only owner

	Male					Female				p-value for interaction
	IRR	LCI	UCI	p value		IRR	CI		p value	
**School Bully**	0.99	0.88	1.12	0.9266		1.01	0.85	1.21	0.8899	0.8718
**Sexual Abuse**	0.87	0.68	1.10	0.2320		0.99	0.82	1.20	0.9279	0.2483
**Physical Abuse**	0.87	0.74	1.03	0.1119		0.85	0.64	1.14	0.2791	0.6146
**Violent Event**	1.01	0.91	1.12	0.8839		1.07	0.91	1.26	0.4273	0.4941
**Witness Trauma**	0.94	0.84	1.06	0.3024		1.04	0.88	1.23	0.6456	0.1920
**Physical Violence btw. Parents**	0.99	0.78	1.26	0.9422		1.08	0.81	1.43	0.6027	0.7151
**Dangerous sch/neighbor**	1.10	0.86	1.42	0.4384		0.71	0.48	1.06	0.0959	0.2316
**Count of violence types mean/sd**	0.95	0.90	1.01	0.0782		1.00	0.93	1.06	0.9166	0.1440
**Adulthood Victim(18+)**										
**Physical assault**	1.00	0.85	1.17	0.9614		1.09	0.91	1.31	0.3650	0.1674
**Sexual assault**	1.08	0.71	1.63	0.7243		1.12	0.92	1.35	0.2470	0.8996

All models adjusted for race, sex, cohort, urbanicity, low SES, and childhood vulnerabilities

© 2023 Caves Sivaraman J et al. *JAMA Network Open.*

eTable 3. Associations between violent experiences and becoming a gun owner in adulthood (Adult-only Owner) versus remaining a Never Owner sensitivity analysis: the "adult-only ownership" category includes people who owned *or had access to* guns in adulthood

Violent Experience	Prevalence: never owners n (%)	Prevalence: adult-only owners n (%)	IRR for adult-only ownership (95% CI)	*P* values
Childhood victimization				
Bullied at school	87 (29.4)	25 (19.6)	0.87 (0.72, 1.05)	0.15
Sexual abuse	51 (13.6)	12 (4.2)	0.80 (0.60, 1.06)	0.12
Physical abuse	41 (11.0)	14 (4.8)	0.92 (0.68, 1.25)	0.61
Violent event	146(54.6)	49(55.3)	1.07 (0.91, 1.27)	0.41
Witness trauma	104 (32.3)	35 (34.2)	1.14 (0.96, 1.36)	0.13
Physical violence between parents	24 (10.9)	9(2.9)	0.82 (0.57, 1.19)	0.29
Dangerous school or neighborhood	38 (9.4)	10 (6.3)	0.86 (0.60, 1.24)	0.43
Count of violence types, mean (standard deviation)	1.53 (1.37)	1.30 (1.19)	0.99 (0.91, 1.07)	0.80
Adulthood victimization				
Physical assault	45 (11.6)	21 (14.7)	1.16 (0.89, 1.52)	0.26
Sexual assault	29 (9.4)	17 (10.8)	1.15 (0.85, 1.55)	0.37

All models adjusted for race, sex, cohort, urbanicity, low SES, and childhood vulnerabilities

© 2023 Caves Sivaraman J et al. *JAMA Network Open.*

eTable 4. Associations between violent experiences and remaining a gun owner in young adulthood (consistent owner) versus being a childhood-only owner sensitivity analysis: the "consistent owners" category includes who owned *or had access to* guns in adulthood

Violent experience	Prevalence: consistent owners n (%)	Prevalence: childhood-only owners n (%)	IRR for consistent ownership (95% CI)	*P* values
Childhood victimization				
Bullied at school	142 (25.4)	123 (26.9)	0.99 (0.91, 1.09)	0.85
Sexual abuse	61 (9.1)	44 (14.6)	0.96 (0.84, 1.09)	0.51
Physical abuse	57 (12.0)	77 (18.9)	0.89 (0.78, 1.02)	0.10
Violent event	230 (53.2)	249(49.2)	1.02 (0.94, 1.10)	0.65
Witness trauma	142 (25.3)	164 (35.0)	0.95 (0.87, 1.21)	0.28
Physical violence between parents	42 (8.3)	29 (6.9)	1.02 (0.86, 1.21)	0.81
Dangerous school or neighborhood	30 (5.2)	50 (7.1)	0.96 (0.79, 1.16)	0.67
Count of violence types, mean (standard deviation)	1.20 (1.13)	1.56 (1.28)	0.97 (0.93, 1.01)	0.12
Adulthood victimization				
Physical assault	85 (15.7)	78 (23.9)	1.01 (0.91, 1.13)	0.80
Sexual assault	37 (9.7)	46 (10.8)	1.07 (0.94, 1.26)	0.25

All models adjusted for race, sex, cohort, urbanicity, low SES, and childhood vulnerabilities

© 2023 Caves Sivaraman J et al. *JAMA Network Open.*
