# Supplementary material for: Violent Experiences and Patterns of Firearm Ownership From Childhood to Young Adulthood
Source: JAMA Netw Open. 2023 Oct 18;6(10):e2336907. doi: 10.1001/jamanetworkopen.2023.36907 (PMC10585415; doi:10.1001/jamanetworkopen.2023.36907)
Supplement: Supplement 2. — Data Sharing Statement [file jamanetwopen-e2336907-s002.pdf]

Data Sharing Statement

Caves Sivaraman. Violent Experiences and Patterns of Firearm Ownership From Childhood to Young Adulthood. *JAMA Netw Open*. Published October 18, 2023.
doi:10.1001/jamanetworkopen.2023.36907

Data
Data available: No

Additional Information
Explanation for why data not available: Access to data from the Great Smokey Mountains Study will be assessed on an individual basis.
